# Genome-Wide Identification and Expression Analysis of Auxin-Responsive *GH3* Gene Family in Pepper (*Capsicum annuum* L.)

**DOI:** 10.3390/plants14142231

**Published:** 2025-07-18

**Authors:** Qiao-Lu Zang, Meng Wang, Lu Liu, Xiao-Mei Zheng, Yan Cheng

**Affiliations:** Shanxi Key Laboratory of Germplasm Resources Innovation and Utilization of Vegetable and Flower, College of Horticulture, Shanxi Agricultural University, Taigu, Jinzhong 030801, China; zangql@sxau.edu.cn (Q.-L.Z.); wm102300@163.com (M.W.); l13593562360@163.com (L.L.); zxm2809968194@163.com (X.-M.Z.)

**Keywords:** pepper, auxin, *Gretchen Hagen 3* gene family, expression pattern

## Abstract

As an auxin-responsive gene, *Gretchen Hagen 3* (*GH3*) maintains hormonal homeostasis by conjugating excess auxin with amino acids in plant stress-related signaling pathways. *GH3* genes have been characterized in many plant species, but the characteristics of pepper (*Capsicum annuum* L.) *GH3* (*CaGH3*) gene family members in response to multiple stimulants are largely unknown. In this study, we systematically identified the *CaGH3* gene family at the genome level and identified eight members on four chromosomes in pepper. *CaGH3*s were divided into two groups (I and III) and shared conserved motifs, domains, and gene structures. Moreover, *CaGH3*s had close evolutionary relationships with tomato (*Solanum lycopersicum* L.), and the promoters of most *CaGH3* genes contained hormone and abiotic stress response elements. A protein interaction prediction analysis demonstrated that the CaGH3-3/3-6/3-7/3-8 proteins were possibly core members of the *CaGH3* family interaction. In addition, qRT-PCR results showed that *CaGH3* genes were differentially expressed in pepper tissues and could be induced by phytohormones (IAA, ABA, and MeJA) and abiotic stresses (salt, low temperature, and drought) with different patterns. In addition, *CaGH3-5* and *CaGH3-7* were cloned, and the sequences showed a high degree of conservation. Moreover, the results of subcellular localization indicated that they were located in the membrane and chloroplast. Notably, after overexpressing *CaGH3-7* in tomato, RNA-seq was performed on wild-type and transgenic lines, and the differentially expressed genes were mainly enriched in response to external stimuli. This study not only lays the foundation for a comprehensive understanding of the function of the *CaGH3* gene family during plant growth and stress responses but also provides potential genetic resources for pepper resistance breeding.

## 1. Introduction

Auxins play important roles in plant growth and development and in responses to biotic and abiotic stress [1,2]. Auxin homeostasis is regulated by several auxin-responsive genes, including *auxin/indoleacetic acid* (*Aux/IAA*), *Gretchen Hagen 3* (*GH3*), and *small auxin up RNA* (*SAUR*), which are considered to be early auxin-responsive gene families [3]. The GH3 protein can combine amino acid with the free form of salicylic acid (SA), jasmonic acid (JA), or indole-3-acetic acid (IAA) to maintain phytohormone homeostasis [4] and then regulate plant growth and development [5,6]. A *GH3* mutant, *ydk1-D*, is involved in hypocotyl elongation through the regulation of auxin activity [7]. In *Capsicum chinense* L., *CcGH3* is involved in fruit ripening through the ethylene pathway [8]. The *AtGH3.9* mutation promotes primary root growth and influences the auxin and jasmonate signaling pathways [9]. Moreover, some *AtGH3* genes can affect the development of lateral roots by modulating the response of auxin and meristem activities [10]. In addition, lines overexpressing *AtGH3.15* have longer roots and lower lateral root density under indolebutyric acid (IBA) treatment, showing resistance to IBA [11]. All in all, *GH3* genes can regulate the growth process of plants through hormones.

In addition to their roles in plant growth, *GH3* family genes also play crucial roles in plant resistance to biotic and abiotic stresses [4,6,12]. Rice (*Oryza sativa* L.) with *OsGH3-8* overexpression enhances resistance to *Xanthomonas oryzae* pv *oryzae* via suppressed auxin signaling [13]. In an experiment where maize (*Zea mays* L.) was treated with *Colletotrichum graminicolum*, most *GH3* genes were down-regulated, but *ZmGH3-2* and *ZmGH3-8* were highly up-regulated [14]. In addition, the activation of *OsGH3.13* enhanced drought tolerance by affecting the concentration of IAA [15], overexpressing *OsGH3-2* reduced the ABA and free IAA level and increased cold tolerance [16], and silencing the *GH3.5* gene in cotton reduced drought and salt tolerance [17]. Moreover, *AtGH3.10* contributes to JA-amino acid biosynthesis and functions in wound stress response [18]. Overall, *GH3*s can also enhance resistance to various stresses by regulating hormones.

Pepper (*Capsicum annuum* L.), which belongs to the Solanaceae family, is an important vegetable crop with high economic benefits [19]. However, the growth of pepper is negatively affected by biotic and abiotic stresses, such as cold, heat, drought, salt, osmotic stress, and disease [20,21,22,23,24]. Endogenous IAA affects the growth and development of pepper plants [25]. Meanwhile, genes related to the auxin signaling pathway can alleviate the damage caused by stress to peppers [26]. *GH3s*, as auxin response genes, play significant roles in the auxin signaling pathway; thus, the study of *CaGH3* of pepper is necessary to determine the biological processes involved in multiple stresses. In this study, we identified the *CaGH3* members in pepper and determined their physicochemical properties, phylogenetic relationships, and expression patterns. The present study not only establishes a foundation for further studies on the pepper *GH3* gene but also provides certain candidate genes with potential applications in molecular breeding to improve pepper stress resistance.

## 2. Results

### 2.1. Identification and Characterization of CaGH3 Gene Family in Pepper

Based on the known GH3 protein domain, a total of eight *CaGH3* gene family members were identified in the pepper genome (Table 1). These *CaGH3* genes were named *CaGH3-1* to *CaGH3-8* based on their chromosome location and were separately located at 4 of the 12 pepper chromosomes—Chr2, Chr7, Chr8, and Chr10, where each chromosome contains two genes (Table 1). Subsequently, the physical and chemical properties of the *CaGH3* genes were predicted, as shown in Table 1. The lengths of these proteins ranged from 575 (CaGH3-5) to 609 (CaGH3-7) amino acids (aa). Their molecular weights (MWs) ranged from 64.28 KDa (CaGH3-5) to 69.42 KDa (CaGH3-7). The lowest isoelectric point (pI) was 5.39 (CaGH3-3) and the highest pI was 7.26 (CaGH3-7). The protein instability coefficients of the CaGH3 proteins ranged from 31.81 (CaGH3-5) to 47.34 (CaGH3-7), and most were unstable, except for two, namely the CaGH3-5 and CaGH3-8 proteins, as their instability indexes were below 40. The lowest predicted hydrophilicity was -0.34 (CaGH3-4) and the highest was −0.11 (CaGH3-8), suggesting that all members of the family were characterized by hydrophilic properties. In addition, the predicted subcellular localization results indicated that eight CaGH3 proteins were located in the chloroplast. The differences in the physical and chemical properties of these members indicated that they might play roles in different biological processes.

**Figure 1:**
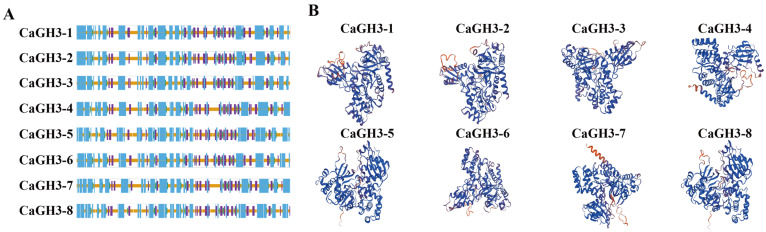
Structural analysis of GH3 family proteins of *Capsicum annuum*. (**A**) Secondary structure analysis of CaGH3 proteins. Blue line, α-helix; green line, β-turn; purple line, extended strand; orange line, random coil. (**B**) Tertiary structure analysis of CaGH3 proteins.

The secondary structure of the CaGH3 proteins consisted of α-helix (41.62–44.35%), β-turn (4.20–5.15%), extended strand (13.06–15.54%), and random coil (36.52–40.83%), among which α-helix and random coil were the main components (Table 1 and Figure 1A). A three-dimensional structural analysis showed that the *CaGH3* gene family members had different structures and indicated their functional diversity (Figure 1B).

### 2.2. Prediction of Collinear Analysis of CaGH3 Family Genes

**Figure 2:**
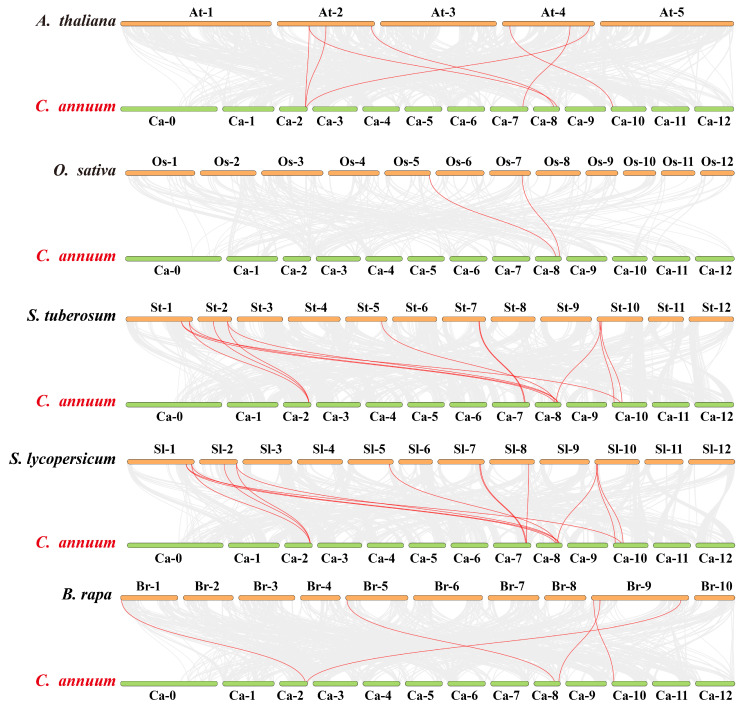
Collinear analysis of *Capsicum annuum* and five other plants (*Arabidopsis thaliana*, *Oryza sativa*, *Solanum tuberosum*, *Solanum lycopersicum*, and *Brassica rapa*). The gray line represents the collinear blocks of the pepper genome and other plant genomes, and the red curve represents the *CaGH3* gene collinearity.

The collinearity relationships of the *GH3* genes between pepper and five other species (*Arabidopsis thaliana*, *Oryza sativa*, *Solanum tuberosum*, *S. lycopersicum*, and *Brassica rapa*) were analyzed (Figure 2). The numbers of homologous pairs in these five species were 7, 2, 13, 14, and 5, respectively, indicating that there were more *GH3* homologous genes between *Solanaceae* crops and pepper. The *CaGH3* genes had the most gene pairs with *S. lycopersicum*. *CaGH3-2* and *CaGH3-5* had three syntenic gene pairs with *S. lycopersicum*; *CaGH3-4*, *CaGH3-6*, and *CaGH3-8* had two pairs; and *CaGH3-3* and *CaGH3-7* had only one pair. Furthermore, we found that *CaGH3-2* had the most syntenic gene pairs: three gene pairs with *S. lycopersicum*, *S. tuberosum*, and *A. thaliana*, respectively, and two gene pairs with *B. rapa*. This suggests that *CaGH3-2* might play a key role in the evolution of the *CaGH3* family. Notably, no homologous genes of *CaGH3-1* were found in these species, indicating that the *CaGH3* genes in different species might have been copied, retained, or lost to different degrees during evolution.

### 2.3. Evolutionary Analysis of CaGH3 Genes Among Multiple Species

**Figure 3:**
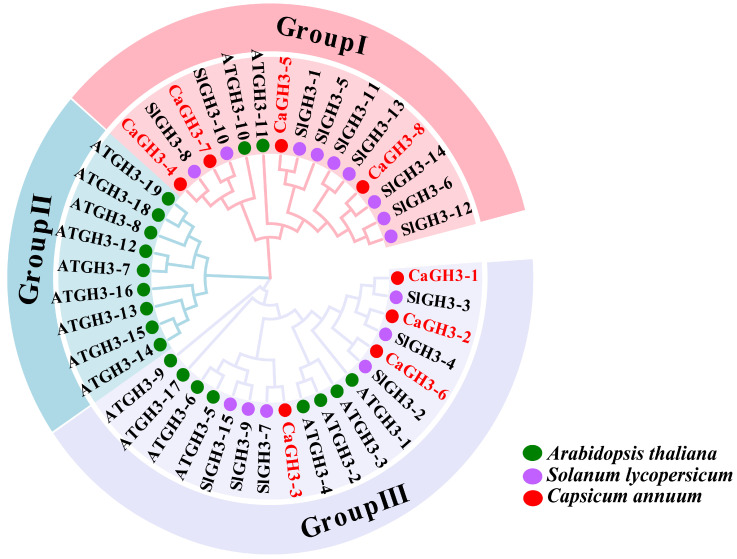
Phylogenetic analysis of GH3 homolog proteins from *Capsicum annuum* (Ca), *Arabidopsis thaliana* (At), and *Solanum lycopersicum* (Sl).

A phylogenetic tree of GH3 proteins from pepper and other two species (*A. thaliana* and *S. lycopersicum*) was constructed (Figure 3). The tree shows that all CaGH3 proteins were divided into two groups (I and III). A total of four CaGH3s (CaGH3-4, CaGH3-5, CaGH3-7, and CaGH3-8) were classified into group I and four CaGH3s (CaGH3-1, CaGH3-2, CaGH3-3, and CaGH3-6) were classified into group III. Interestingly, there were no CaGH3 members clustered into group II. These results indicate that the functions of CaGH3s in pepper are diverse.

### 2.4. Phylogenetic, Conserved Motifs, Domains, and Gene Structures of CaGH3 Family Genes

**Figure 4:**
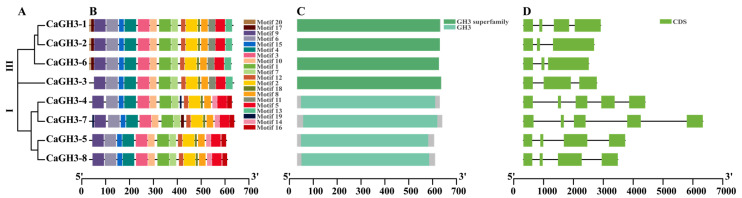
Phylogenetic relationships (**A**), conserved motifs (**B**), conserved domains (**C**), and gene structures (**D**) of CaGH3s.

To further predict the function of CaGH3 proteins, 20 conserved motifs were identified using MEME (Figure 4B). The number of motifs of CaGH3 proteins varied from 15 to 17, and all of them contained 13 conserved motifs. However, some conserved motifs were specific to the group classification. Motifs 14 and 16 were only presented in group I and motifs 11 and 13 were only presented in group III. Thus, these motifs might play different functions in different groups (Figure 4A,B). Furthermore, the analysis of the conserved domains showed that CaGH3 protein sequences all contain conserved GH3 domains or GH3 superfamily domains (Figure 4C). The exon/intron structures of *CaGH3*s were determined by comparing their genomic DNA sequences (Figure 4D). The results show that all of the coding sequences of the *CaGH3* genes were disrupted by introns; the number of introns ranged from two to four and the number of exons ranged from three to five. Generally, the numbers and lengths of exons and introns were specific for each group (Figure 4D). Overall, the conserved motif, domains, and gene structures implied the similar function of the CaGH3 members.

### 2.5. Analysis of Cis-Acting Elements of CaGH3 Promoters

**Figure 5:**
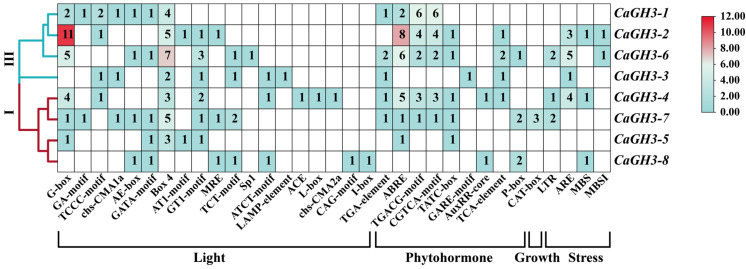
Cis-regulatory element analysis of *CaGH3* family genes. The number of each cis-acting element is shown in the heatmap box, ranging from blue to red, with white boxes indicating that there are no corresponding cis-acting elements.

The types and numbers of the cis-acting elements in the promoter sequences of *CaGH3* genes were analyzed (Figure 5). In total, four kinds of cis-acting elements, including light-responsive elements, phytohormone-responsive, growth-related, and stress-related elements were randomly distributed in the promoter sequences [27,28,29]. The number of MeJA-responsive elements (TGACG motif and CGTCA motif) was the largest (32), followed by the ABA-responsive elements (ABRE, 23), auxin-responsive elements (TGA-element, AuxRR-core, 8), SA-responsive elements (TCA-element, 5), gibberellin-responsive elements (GARE-motif, P-box, TATC-box, 11), low-temperature-responsive elements (LTR, 5), and drought induction elements (MBS, 3). Notably, 50% of the promotors of *CaGH3* genes contained anaerobic induction elements (AREs) and some had flavonoid biosynthesis elements (MBSI, 2). Overall, these elements were present in the promoters of the *CaGH3* genes, implying that *CaGH3* genes are widely involved in plant responses to various stimuli.

### 2.6. Expression Analysis of CaGH3 Genes in Different Tissues

**Figure 6:**
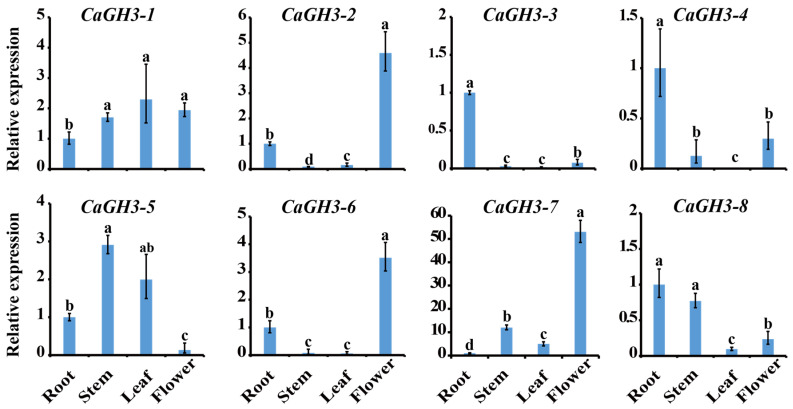
Expression patterns of *CaGH3* gene family in different tissues. All data points are means ± standard errors. Different lowercase superscripts indicate significant differences, as determined using Duncan’s new multiple range test (*p*-value < 0.05).

The expressions of *CaGH3* genes in different tissues (root, stem, leaf, and flower) were detected via qRT-PCR (Figure 6). The expression level of *CaGH3-1* was the lowest in the root and showed no significant differences in the other tissues. The transcript levels of *CaGH3-2* and *CaGH3-6* were found to be hardly detectable in the stem and leaf, while they showed lower expression levels in the root and higher expression levels in the flower. The expression levels of *CaGH3-3* and *CaGH3-4* were high in the root, while low in the other tissues. The transcript level of *CaGH3-5* was hardly detectable in the flower, but easily detectable in the other tissues, while the expression level of *CaGH3-7* was highest in the flower compared to in the other tissues. The transcript level of *CaGH3-8* was higher in the root and stem than in the other tissues. In short, all *CaGH3* genes were differentially expressed in various tissues, indicating that their functions occurred at different stages of development.

### 2.7. Expression Patterns of CaGH3 Genes in Response to Different Hormone Treatments

**Figure 7:**
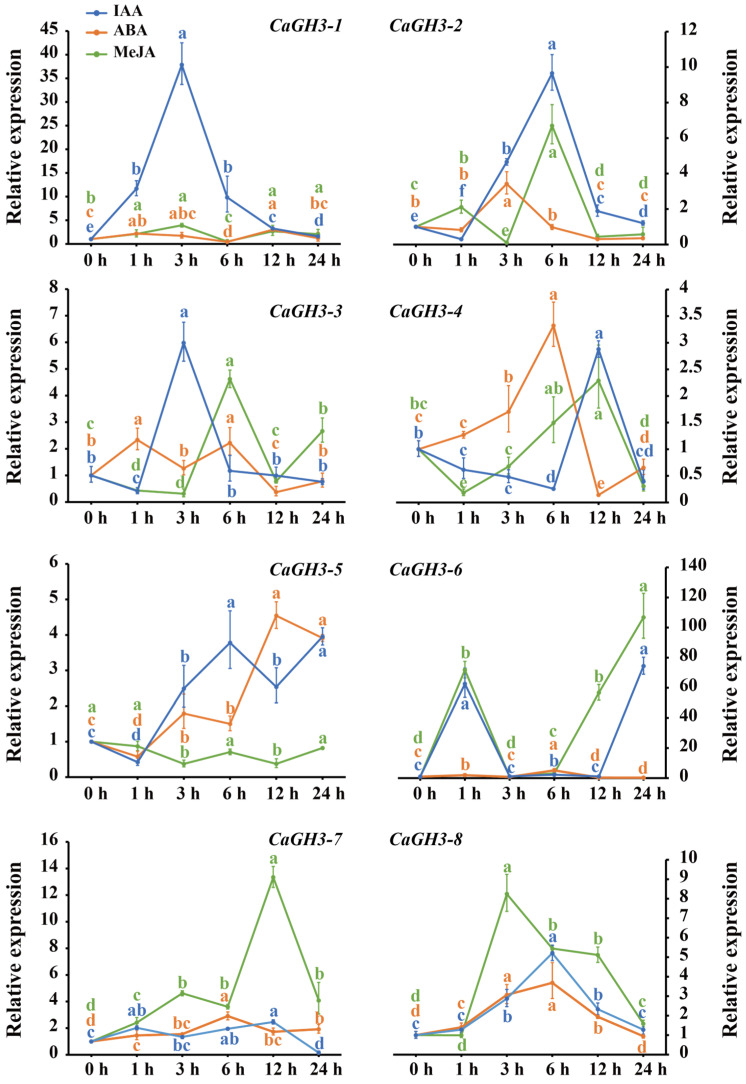
Expression patterns of *CaGH3* gene family under different hormone treatments. All data points are means ± standard errors. Different lowercase superscripts indicate significant differences, as determined using Duncan’s new multiple range test (*p*-value < 0.05).

The expression patterns of *CaGH3* genes under IAA, ABA, and MeJA treatments were analyzed (Figure 7). Under the IAA treatment, the expressions of *CaGH3-1* and *CaGH3-8* exhibited a pattern of initial increase and subsequent decrease, and significant up-regulation after 3 h (37.84-fold) and 6 h (5.21-fold), respectively. The expression of *CaGH3-2*, *CaGH3-3*, and *CaGH3-4* first declined, then rose, and finally dropped to a relatively low level. Among them, *CaGH3-2* reached its peak expression at 6 h (9.65-fold) after treatment; *CaGH3-3* at 3 h (5.98-fold); and *CaGH3-4* at 12 h (2.88-fold). Interestingly, the expression of *CaGH3-5* first declined, then rose, showing a fluctuating trend, and finally rose to the highest level at 24 h after treatment (3.95-fold). Moreover, the expression level of *CaGH3-7* increased first, then remained at a relatively high level within 12 h after treatment, and finally decreased after 12 h. Notably, the expression level of *CaGH3-6* was very high at 1h (62.57-fold) and 24 h (74.42-fold) after treatment but hardly detectable at other times.

Under the ABA treatment, all *CaGH3* members except *CaGH3-1*, *CaGH3-3*, and *CaGH3-5* exhibited a pattern of initial increase and subsequent decrease. Among them, *CaGH3-2* reached its peak expression at 3 h (3.42-fold) after treatment, with *CaGH3-4* (3.32-fold), *CaGH3-6* (5.15-fold), *CaGH3-7* (2.9-fold), and *CaGH3-8* (3.69-fold) at 6 h. Moreover, the expression levels of *CaGH3-1* and *CaGH3-3* first increased, then showed a fluctuating trend, and finally reached their peak at 12 h (3.00-fold) and 1 h (2.33-fold), respectively. Additionally, the expression of *CaGH3-5* exhibited a pattern of initial decrease and subsequent increase after treatment.

Under the MeJA treatment, the expression levels of *CaGH3-1* and *CaGH3-2* first increased, then showed a fluctuating trend, and finally reached their peak at 3 h (3.93-fold) and 6 h (6.69-fold), respectively. Meanwhile, the expression levels of *CaGH3-3, CaGH3-4,* and *CaGH3-5* first decreased and then showed a fluctuating trend. Interestingly, the expression of *CaGH3-6* increased sharply at 1 h (71.81-fold) after treatment and then dropped quickly to the lowest level, finally reaching its highest level at 24 h (106.79-fold). Notably, *CaGH3-7* and *CaGH3-8* exhibited a pattern of initial increase and subsequent decreased, and they reached the peak at 12 h (13.34-fold) and 3 h (8.24-fold), respectively.

In summary, the expression levels of *CaGH3-1*, *CaGH3-2*, and *CaGH3-3* were relatively high after IAA treatment; those of *CaGH3-4* and *CaGH3-5* were relatively high after ABA treatment; and those of *CaGH3-6*, *CaGH3-7*, and *CaGH3-8* were relatively high after MeJA treatment. These results reveal that all *CaGH3* genes might play regulatory roles in these signaling pathways.

### 2.8. Expression Patterns of CaGH3 Genes in Response to Different Stresses

**Figure 8:**
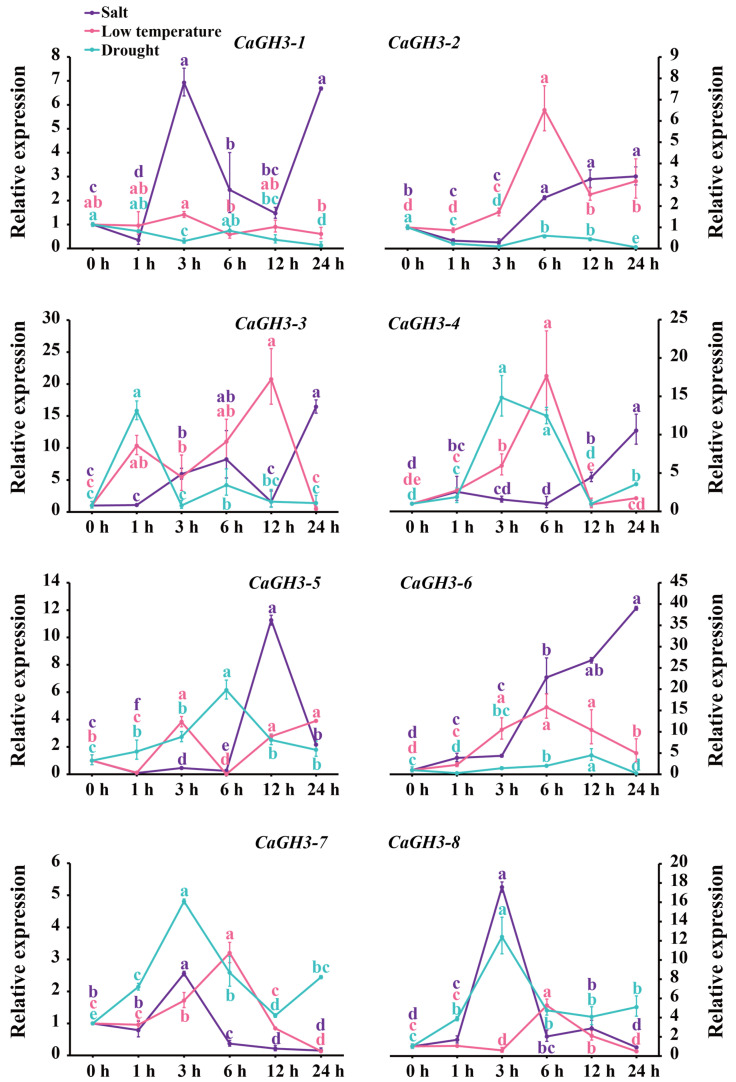
Expression patterns of *CaGH3* gene family under different abiotic stresses. All data points are means ± standard errors. Different lowercase superscripts indicate significant differences, as determined using Duncan’s new multiple range test (*p*-value < 0.05).

The expression patterns of *CaGH3* genes under salt, low-temperature, and drought treatments were analyzed (Figure 8). Under the salt treatment, the expression level of *CaGH3-1* and *CaGH3-5* first decreased, then increased to a peak at 3 h (6.93-fold) and 12 h (11.26-fold), and then dropped again, while that of *CaGH3-1* increased again at 24 h. Moreover, *CaGH3-2* exhibited a pattern of initial decrease and then an increase to the peak at 24 h (3.39-fold). Conversely, *CaGH3-7* (2.56-fold) and *CaGH3-8* (17.56-fold) showed a pattern of initial increase to their peak at 3 h and then a drop to the lower level. The expression of *CaGH3-3* first increased, then declined, and finally reached its peak at 24 h (16.44-fold). Notably, the expression levels of *CaGH3-4* (10.53-fold) and *CaGH3-6* (39.00-fold) gradually increased and reached their peak at 24 h.

Under the low-temperature treatment, all *CaGH3* members except for *CaGH3-1* and *CaGH3-5* exhibited a pattern of initial increase and subsequent decline. Among them, *CaGH3-2* (6.51-fold), *CaGH3-4* (17.64-fold), *CaGH3-6* (15.77-fold), *CaGH3-7* (3.20-fold), and *CaGH3-8* (5.27-fold) reached their peak at 6 h after treatment, with *CaGH3-3* reaching it at 12 h (20.74-fold). The expression of *CaGH3-5* first decreased at 1 h, then increased at 3 h (3.83-fold), and then dropped quickly at 6 h, finally reaching a high level at 24 h (3.90-fold). Notably, the expression of *CaGH3-1* did not change significantly in response to low temperature.

Under the drought treatment, all *CaGH3* genes except for *CaGH3-1*, *CaGH3-2*, and *CaGH3-6* showed a trend of increasing at first and then decreasing. Among them, the expressions of *CaGH3-4* (14.83-fold), *CaGH3-7* (4.81-fold), and *CaGH3-8* (12.41-fold) reached their peak at 3 h after treatment, with *CaGH3-3* and *CaGH3-5* reaching the peak at 1 h (15.82-fold) at 6 h (6.15-fold), respectively. In addition, the expression of *CaGH3-1*, *CaGH3-2*, and *CaGH3-6* first declined, then increased, and finally dropped to a low level.

Overall, the expression levels of *CaGH3-1*, *CaGH3-5*, and *CaGH3-6* were relatively high under salt treatment; those of *CaGH3-2* and *CaGH3-3* were relatively high under low-temperature treatment; and that of *CaGH3-7* was relatively high under drought treatment. These results reveal that all *CaGH3* genes might play regulatory roles in these signaling pathways.

### 2.9. Prediction of Interacting Proteins Among CaGH3 Family Genes

**Figure 9:**
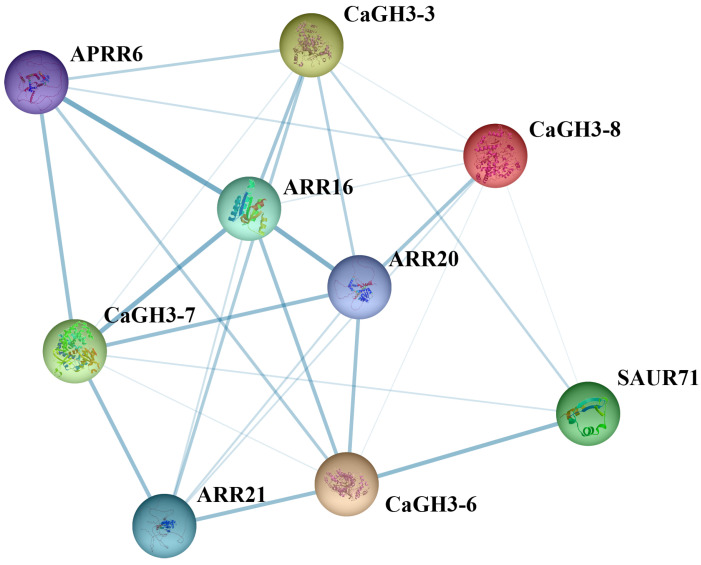
The protein interaction network of pepper *CaGH3* family members. The nodes in the protein interaction network indicate all proteins produced by a protein-coding locus, and different colors indicate different degrees of interaction. The deeper the color of the lines between the nodes, the higher the intensity of the interaction. The thinner lines indicate weaker interactions between the proteins.

To deeply comprehend the molecular mechanism of CaGH3s, the interactions among eight CaGH3 proteins were examined (Figure 9). In total, nine nodes and 28 edges were determined, indicating that nine proteins have 28 interactions, including four CaGH3 proteins. Among them, the CaGH3-3, CaGH3-6, CaGH3-7, and CaGH3-8 proteins interacted with two pepper CaGH3 members, indicating that they were possibly core members of the CaGH3 family. Furthermore, in addition to the CaGH3 family, CaGH3-3 and CaGH3-6 both had strong interaction relationships with five proteins: Small auxin up RNA 71 (SAUR71), *Arabidopsis* response regulator 16 (ARR16), ARR20, ARR21, and *Arabidopsis* putative response regulator-like 6 (APRR6); CaGH3-7 had strong interactions with four proteins: ARR16, ARR 20, ARR 21, and APRR6; and CaGH3-8 had strong interactions with two proteins: ARR 20 and APRR6. Therefore, this protein interaction network analysis offered evidence for verifying the function and mechanism of CaGH3 proteins.

### 2.10. Cloning and Sequence Analysis of CaGH3-5 and CaGH3-7

**Figure 10:**
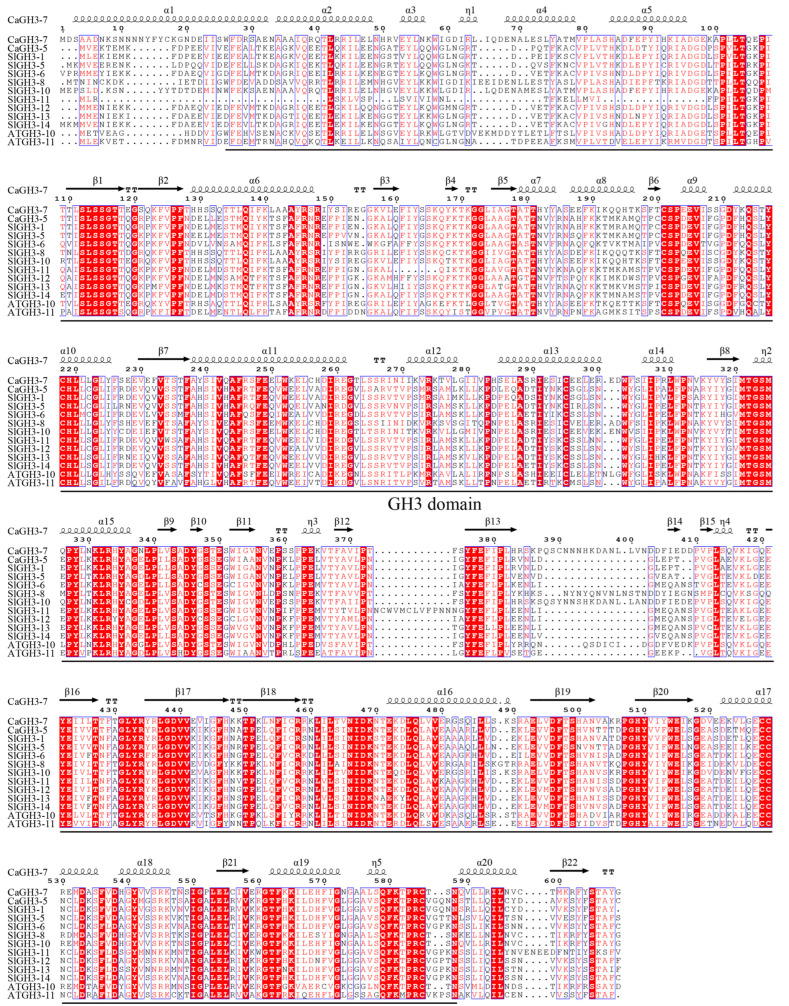
Multiple alignments of GH3 proteins for *C. annuum* (Ca), *S. lycopersicum* (Sl), and *A. thaliana* (At). Conserved residues are highlighted with red boxes, with similar residues shown in a lighter color. The underlying amino acid sequence indicates the GH3 domain.

In order to verify the protein structure of the *GH3* gene family, *CaGH3-5* and *CaGH3-7*, belonging to group I, were cloned. The full length of the coding sequence of *CaGH3-5* (GenBank accession number, PV577549) was 1728 bp, encoding 575 amino acids, and that of *CaGH3-7* (GenBank accession number, PV577548) was 1830 bp, encoding 609 amino acids. Multiple sequence alignments revealed that the amino acid sequence of CaGH3-5 and CaGH3-7 had high similarity with the members of the same group in *S. lycopersicum* and *A. thaliana* (Figure 10). In addition, these proteins all shared the conserved GH3 domain and conserved structures of β1, α9, η2, α15, β10, β20, and β21, revealing why they belong to the same group (Figure 10).

### 2.11. CaGH3-5 and CaGH3-7 Are Membrane and Chloroplast Localization Proteins

**Figure 11:**
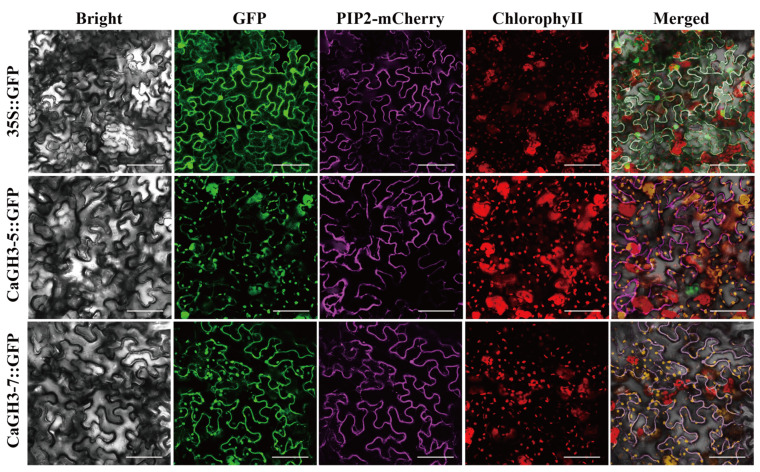
Subcellular localization of CaGH3-5 and CaGH3-7 in *Nicotiana benthamiana*. GFP fluorescence was observed with a fluorescence microscope. PIP2-mCherry was used for membrane localization. Images were taken in a dark field for green fluorescence, while the cell outlines were photographed in a bright field. Bars = 25 µm.

Sequence prediction indicated that the members of the *CaGH3* gene family were located in chloroplasts. To further verify the locations of CaGH3-5 and CaGH3-7, a subcellular localization analysis was conducted in tobacco using an *Agrobacterium*-mediated method. As shown in Figure 11, CaGH3-5 and CaGH3-7 were both located in the membrane and chloroplast, indicating their potential functions.

### 2.12. Transcriptome Analysis of Tomato with Overexpression of CaGH3-7

To further investigate the mechanisms underlying the regulation of *CaGH3* by hormones and stress, *CaGH3-7* was overexpressed in tomato (*Solanum lycopersicum* L.). Then, transcriptome sequencing and differential gene expression analysis were carried out. In transgenic plants, a total of 915 genes were down-regulated and 611 were up-regulated (Supplementary Table S1), which were used to perform Gene Ontology (GO) and Kyoto Encyclopedia of Genes and Genomes (KEGG) pathway analysis.

**Figure 12:**
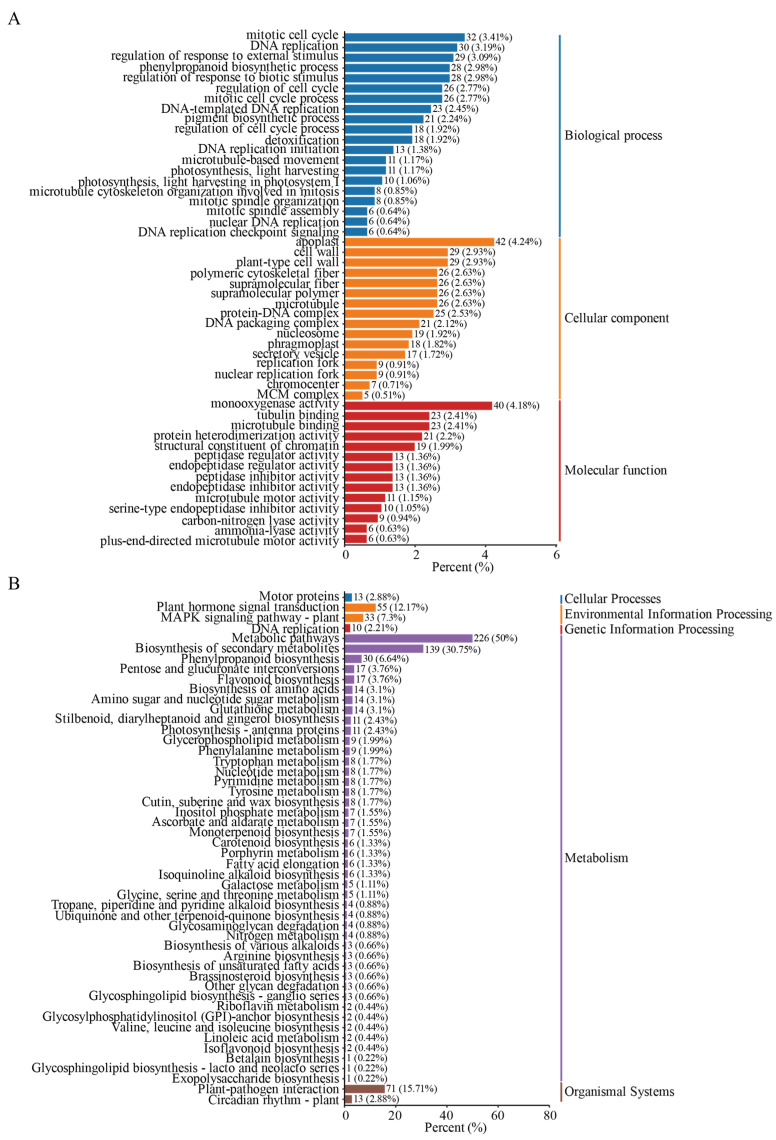
Transcriptome-based enrichment analysis of differentially expressed genes. (**A**) Gene Ontology (GO) classification. (**B**) Kyoto Encyclopedia of Genes and Genomes (KEGG) classification. The numbers after the bars indicate the number and percentage of genes annotated.

For the GO classification analysis of DEGs, 50 sub-categories from three main GO functional categories were selected (Figure 12, Supplementary Table S2). In biological processes, a maximum of 32 genes responded to mitotic cell cycle, followed by DNA replication (30 genes), the regulation of the response to external stimulus (29 genes), the phenylpropanoid biosynthetic process (28 genes), and the regulation of the response to biotic stimulus (28 genes). In terms of cellular components, the top three were apoplast, cell wall, and plant-type cell wall, with 42, 29, and 29 genes, respectively. In addition, at the molecular level, the top three were monooxygenase activity, tubulin binding, and microtubule binding, with 40, 23, and 23 genes, respectively.

Through KEGG analysis (Figure 12, Supplementary Table S3), the top 50 most significantly enriched pathways could be classified into five categories: cellular processes, environmental information processing, genetic information processing, metabolism, and organismal systems. In environmental information processing, two pathways were enriched: plant hormone signal transduction (55 genes) and mitogen-activated protein kinase (MAPK) signaling pathway—plant (33 genes). In terms of metabolism, the top two pathways were metabolic pathways (226 genes) and the biosynthesis of secondary metabolites (139 genes). In addition, regarding organismal systems, plant–pathogen interaction (71 genes) and circadian rhythm—plant (13 genes) were enriched.

## 3. Discussion

When pepper plants are exposed to unsuitable environments such as those with high salt, drought, and extreme temperature, they will stop growing and developing, and may even die [20,21,23]. Therefore, it is particularly important to explore the molecular mechanisms that can alleviate stress injury. Auxin plays a very important role in the growth and development of plants as well as their resistance to stress [30]. *GH3* genes, as early response genes of auxin, can alleviate the damage of stress to plants by regulating hormone homeostasis [31,32]. The identification of *GH3* members is of great significance for the study of pepper stress resistance.

In the present study, eight *CaGH3* members in pepper were identified through bioinformatics. The number of *CaGH3* members was lower than that of *Arabidopsis* (19 members) and tomato (15 members), which might be due to the quality of genome assembly, the loss of genes during evolution, etc. [33]. CaGH3 proteins were classified into two categories (I and III) (Figure 3), and it was the same with SlGH3 and StGH3 proteins, which might be because they all belong to the Solanaceae family [29,33]. Meanwhile, in *Arabidopsis*, GH3 proteins were classified into three groups (I, II, and III) [6]. The differences in the grouping of GH3 proteins might be related to gene retention and loss and functional redistribution among different species during evolution [34]. The genes in groups I and II were reported to be related to the JA signaling pathway [35,36] and growth hormone regulation [16], respectively. In addition, the group III *GH3* gene, *AtPBS3,* regulated SA-dependent defense responses [37]. Thus, we speculated that pepper *CaGH3* members might play a more significant role in resisting stress.

The differences in protein and gene structures also affect the diversity of functions [38,39]. All CaGH3 proteins contained conserved GH3 domains or GH3 superfamily domains, but they had different types of motifs and gene structures (Figure 4). All CaGH3 proteins contained 13 conserved motifs; however, the motifs varied among members of different groups. Motifs 14 and 16 were only present in group I and motifs 11 and 13 were only present in group III. In addition, the numbers of exons and introns were different and specific within each group. Notably, the length of introns in group I was longer than that in group III. These results were consistent with those of potato (*Solanum tuberosum* L.) *GH3* members [29] in that the different groups of *CaGH3* members indicate the different biological functions they may have.

The elements in the promoter can indirectly reflect the potential regulatory patterns of genes [39]. The existence of auxin-responsive elements in GH3 members was important for their response to auxin [40]. Auxin-related elements (TGA-element, AuxRR-core) were found in 75% (six out of eight) of the *CaGH3* gene family members. This suggests that *CaGH3s* could play important roles in auxin regulation [29,40]. Moreover, the promoters of *CaGH3* members contained lots of GA-related elements (GARE-motif, P-box, TATC-box, six out of eight) and ABA-related elements (ABRE, six out of eight), which were reported to be related to growth and stress [28,41]. In addition to response to hormones, *GH3* members were also involved in biotic and abiotic stress responses [6,29]. Likewise, in the pepper, many stress-related regulatory elements were found in the promoters of *CaGH3* genes, such as MeJA-responsive (TGACG-motif, CGTCA-motif, five out of eight), SA-responsive (TCA elements, four out of eight), low-temperature-responsive (LTR, three out of eight), drought-responsive (MBS, three out of eight), and flavonoid biosynthesis elements (MBSI, two out of eight), indicating *CaGH3* members might be sensitive to multiple stresses.

The location of gene expression is very important for the prediction of its function. In potato, *StGH3.2*, *StGH3.3*, and *StGH3.7* showed high levels in the roots, tubers, and flowers, respectively [29]. In tomato, most *GH3* genes had high expression levels in the leaves and cotyledons [33]. In wheat (*Triticum aestivum* L.), *GH3* genes had high expression levels in the leaves and roots [38], while in apple (*Malus* × *domestica*), the expression levels of most *MdGH3* genes in the leaves were much lower than those in the roots [42]. Likewise, in alfalfa (*Medicago sativa* L.), most *GH3* members showed lower levels in leaves than in other organs [43]. Notably, *GH3* genes in maize showed higher levels in the stem [44], while *GH3* genes in rice showed higher levels in flowers [45]. In the present study, *CaGH3-2*, *CaGH3-6*, and *CaGH3-7* were mainly expressed in the flowers, and *CaGH3-3*, *CaGH3-4*, and *CaGH3-8* were mainly expressed in the roots (Figure 6), indicating that they may perform different functions during pepper growth and development.

The cis-acting elements in the promoter predict that the *GH3* genes can respond to lots of hormones [14,15]. In pepper, hormone-responsive elements also occurred in the promoter of *CaGH3* genes, indicating that their expression was regulated by these factors. Under IAA treatment, the expression of *CaGH3-1*, *CaGH3-6*, *CaGH3-7*, and *CaGH3-8* increased first, while that of *CaGH3-2*, *CaGH3-3*, *CaGH3-4*, and *CaGH3-5* decreased first. Under ABA treatment, the expression of *CaGH3-5* decreased first, while that of other members increased first. Under MeJA treatment, the expression of *CaGH3-3*, *CaGH3-4*, and *CaGH3-5* decreased first, while that of other members increased first. Taken together, when the pepper was treated with IAA, ABA, and MeJA, *CaGH3-6*, *CaGH3-7*, and *CaGH3-8* were up-regulated, while *CaGH3-5* was down-regulated. This indicates that these genes mediated the crosstalk between auxin and other hormones. As reported in maize, some *GH3* members were up-regulated, while some were down-regulated when treated with IAA [44]. Most *StGH3* members were up-regulated under ABA and MeJA treatment [29]. Notably, *CaGH3-1*, *CaGH3-2*, and *CaGH3-3* might be more sensitive to IAA; *CaGH3-4* and *CaGH3-5*, to ABA; and *CaGH3-6*, *CaGH3-7*, and *CaGH3-8*, to MeJA. Therefore, different groups of *GH3* genes, and even different members in the same group, could show various expression patterns under hormone treatment, exhibiting the diversity of functions.

More importantly, GH3 members were also reported to play important roles in response to stress [6,13]. Likewise, the promoter of *CaGH3* genes contained lots of stress-responsive elements, indicating these genes were regulated by stress. Under salt treatment, the expression of *CaGH3-1*, *CaGH3-2*, and *CaGH3-5* decreased first, while that of other members increased first. Under low temperature treatment, the expression of *CaGH3-5* decreased first, while that of other members increased first. Under drought treatment, the expression of *CaGH3-1*, *CaGH3-2*, and *CaGH3-6* decreased first, while that of other members increased first. Taken together, when the pepper was treated with salt, low temperature, and drought, *CaGH3-3*, *CaGH3-4*, *CaGH3-7*, and *CaGH3-8* were all up-regulated, indicating that these genes might play important roles in alleviating abiotic stress in pepper. These results also indicate that some genes could be induced by multiple stresses, but some genes exhibited different expression patterns under different stresses. Interestingly, the expression patterns of *CaGH3* genes within the same group were either the same or different. In maize, *ZmGH3-1* was up-regulated under cold; *ZmGH3-9*, under heat; and *ZmGH3-2*, under salt [14]. In rice, *OsGH3.13* was induced by drought [15], and *OsGH3-2* was induced by drought but suppressed by cold; seedlings with overexpression of *OsGH3-2* showed reduced ABA, free IAA levels, and sensitivity to drought [16]. In cotton, the expression levels of most *GH3* genes were enhanced under drought and salt [17]. In potato, *StGH3.2* and *StGH3.6* responded quickly to low temperatures, and *StGH3.3* was more sensitive to salt treatment [29]. Notably, *CaGH3-1*, *CaGH3-5*, and *CaGH3-6* might be more sensitive to salt; *CaGH3-2* and *CaGH3-3* to low temperature; and *CaGH3-7* to drought. In brief, different *CaGH3* genes responded differently to stress, indicating that the functions of genes in specific biological processes should be further identified.

To further identify the structure and function of *CaGH3s*, *CaGH3-5 and CaGH3-7* were cloned and subjected to sequence analysis, which revealed that they contained a conserved GH3 domain and exhibited high sequence homology with tomato protein sequences. The expression location of the genes is crucial for the study of its functions. In *Saccharum*, ScGH3-1 is located in the cell membrane and nucleus [6], while in rice, OsGH3-5 is located in the endoplasmic reticulum, matching the reduction in the free auxin contents in *OsGH3-5* overexpressing plants [46]. In this study, CaGH3-5 and CaGH3-7 were both expressed in the membrane and chloroplast. They were located on the cell membrane, which might make them receive hormone signal molecules, thereby participating in the growth and development process of plants [6]. In addition, by regulating chloroplast movements, auxin is involved in lots of physiological processes, such as phototropic bending and stomatal movement [47]. The CaGH3 proteins, as auxin response proteins, were located in chloroplasts, indicating that they might regulate plant physiological responses by influencing chloroplast movements. In short, the homologous genes in different species showed various characteristics, suggesting the diversity of their functions.

In addition, after overexpressing *CaGH3-7* in tomato and performing transcriptome sequencing, an enrichment analysis of differentially expressed genes was conducted. GO annotation revealed that it participated in the following major biological processes: regulating the response to external stimulus, the phenylpropanoid biosynthetic process, and regulating the response to biotic stimulus. KEGG pathway analysis revealed that the major pathways it was involved in included plant hormone signal transduction, MAPK signaling pathway—plant, the biosynthesis of secondary metabolites, and plant–pathogen interaction. These results explain that the *CaGH3* gene family could indeed respond to exogenous hormone and stress stimuli, which is consistent with previous results [13,17,29].

Considering that *CaGH3* members play important roles in alleviating stress, the exploration of its genetic mechanism is helpful for further molecular breeding. The probable interacting proteins of CaGH3 members were predicted, and the results showed that some of the members would interact with proteins SAUR71, ARR16, ARR20, ARR21, and APRR6, which play important roles in the auxin and cytokinin transduction pathways. The *Arabidopsis SAUR41* subfamily genes, containing *SAUR40*, *SAUR41*, *SAUR71*, and *SAUR72*, can be induced with ABA to modulate cell expansion and salt tolerance [48]. The inducible expression of *Arabidopsis ARR22* in transgenic plants enhanced drought and freezing tolerance by enhancing cell membrane integrity [49]. Taken together, these results indicate that the *CaGH3s* might alleviate stress injury by participating in the hormone regulation pathway. Notably, based on the results, we can screen out genes related to the biological processes of concern, obtain the plants with overexpression and silenced expression, and identify their functions by observing the phenotypes. Furthermore, the pepper genetic transformation system could be utilized to obtain novel pepper germplasm resources, and comprehensive evaluations of transgenetic plants would be conducted, thereby achieving pepper resistance breeding.

## 4. Materials and Methods

### 4.1. Identification of the CaGH3 Gene Family

The genome data of the *Capsicum annuum* Zunla database (v2.0) were downloaded from the Plant GARDEN (https://plantgarden.jp/en/list/t4072/genome/t4072.G002, accessed on 25 August 2023). The Hidden Markov Model (HMM) profiles of the GH3 domain (PF 03321) were downloaded from the InterPro database (https://www.ebi.ac.uk/interpro/, accessed on 23 July 2024) [50]. Then, the *CaGH3*s were searched in the pepper database using HMMER3.0 (E < 1 × 10^−10^) [51]. Meanwhile, based on the genome data of the *Capsicum annuum*, blastp was conducted with 19 *A. thaliana* GH3s (AtGH3s) from the Arabidopsis Information Resource (TAIR10) database (https://www.arabidopsis.org/index.jsp, accessed on 3 March 2022) to screen the CaGH3 proteins (E < 1 × 10^−10^) [52]. Based on the above two methods, the candidate proteins were identified using the InterPro and SMART (http://smart.embl-heidelberg.de/, accessed on 20 July 2024) databases to determine whether they contained the GH3 domain.

### 4.2. Sequence Features and Structures of CaGH3 Gene Family

The physicochemical properties of pepper CaGH3 proteins were analyzed using the ExPASy server 10 (https://prosite.expasy.org/, accessed on 21 July 2024) [53]. Plant-mPLoc (http://www.csbio.sjtu.edu.cn/bioinf/plant-multi/, accessed on 23 July 2024) was used to predict the subcellular localization of the CaGH3 proteins. In addition, SPOMA (https://npsa.lyon.inserm.fr/cgi-bin/npsa_automat.pl?page=/NPSA/npsa_sopma.html, accessed on 18 April 2024) and SWISS-MODEL (https://swissmodel.expasy.org/, accessed on 21 July 2024) were used to predict their secondary and tertiary structures.

### 4.3. Chromosomal Location and Collinearity Analysis of the CaGH3 Gene Family

The chromosome locations of the *CaGH3*s were obtained from the pepper database. Mappings of the physical locations of the *CaGH3*s on pepper chromosomes were drawn with TBtools [54]. The genomic sequence and GFF annotation files of five species (*A. thaliana*, *O. sativa*, *S. tuberosum*, *S. lycopersicum*, and *B. rapa*) were downloaded from EnsemblPlants (http://plants.ensembl.org/index.html, accessed on 9 July 2022). The collinearity of the pepper *CaGH3* genes and the five species was analyzed using TBtools (v. 2.104) [54].

### 4.4. Phylogenetic Analysis of the CaGH3 Gene Family

A total of 19 *A. thaliana* GH3s (AtGH3s) from the *Arabidopsis* data and 15 *S. lycopersicum* GH3s (SlGH3s) from their genomic data were downloaded, respectively (Supplementary Table S4). Then, phylogenetic analysis was performed by aligning all GH3 protein sequences using the ClustalX 1.83 program and an un-rooted neighbor-joining phylogenetic tree [55,56].

### 4.5. Conserved Motifs, Domains, and Gene Structures of the CaGH3 Gene Family

The conserved motifs of the CaGH3 proteins were analyzed using MEME5.2.0 software (https://meme-suite.org/meme/doc/download.html, accessed on 24 July 2024). The number of different motifs was 20 and the motif length ranged from 6 to 50 amino acids [57] (Supplementary Table S5). Their conserved domains were analyzed in NCBI CDD (https://www.ncbi.nlm.nih.gov/Structure/cdd/wrpsb.cgi, accessed on 20 July 2024). GSDS2.0 software (https://gsds.gao-lab.org/Gsds_about.php, accessed on 3 June 2022) was used to analyze the gene structures of the *CaGH3* genes [58].

### 4.6. Cis-Acting Regulatory Element Analysis of CaGH3 Gene Promoters

The 2000 bp sequences upstream of the start codon of the *CaGH3* genes were considered promoters, and the cis-acting elements of CaGH3s were predicted using the PlantCARE server (http://bioinformatics.psb.ugent.be/webtools/plantcare/html/, accessed on 24 July 2024) [27]. Then, the results were visualized with TBtools [54].

### 4.7. Plant Materials, Growth Conditions, and Hormone and Stress Treatments

Pepper seeds were germinated in a light incubator at 28 °C. Five days later, the germinated seeds were transplanted into pots containing soil/vermiculite/perlite (2:1:1) and placed in a growth chamber under long-day conditions (16 h^−1^ light/8 h^−1^ dark, 23/20 °C day/light, 150 µmol·m^−2^·s^−1^). Six-leaf seedlings were treated with 100 μM abscisic acid (ABA), 100 μM methyl jasmonate (MeJA), 100 μM indoleacetic acid (IAA), 20% polyethylene glycol-6000 (PEG-6000), and 200 mM NaCl. A low temperature was applied by placing the seedlings in a 4 °C growth chamber. The leaf tissues were harvested at 0, 1, 3, 6, 12, and 24 h after various treatments. Additionally, samples of the root, stem, leaf, and flower were harvested to investigate the tissue-specific expressions. All these samples were collected in triplicate, directly frozen in liquid nitrogen, and stored at −80 °C until RNA extraction and qRT-PCR.

### 4.8. RNA Extraction, qRT PCR, and Statistical Analysis

Total RNA was extracted using the RNAiso Plus reagent kit (TaKaRa, Shiga, Japan) according to the manufacturer’s instructions. The quality of the RNA samples was verified using agarose gel electrophoresis. A total of 2 μg of RNA of each sample was used for first-strand cDNA synthesis using M-MLV reverse transcriptase according to the manufacturer’s protocols (TransGen, Beijing, China). The specific primers of *CaGH3*s and the internal reference gene (*CaUBI3*; GenBank accession number, AY486137.1) are shown in Supplementary Table S6.

qRT-PCR was carried out using TB Green^®^ Premix Ex Taq™ II (TaKaRa, Shiga, Japan) according to the manufacturer’s instructions. Triplicate qRT-PCR experiments were performed for all samples, and the data were shown as the means ± SDs. Statistical analysis was performed with SPSS 21.0 using analysis of variance.

### 4.9. Protein Interaction Prediction Analysis of the CaGH3 Gene Family

To investigate the CaGH3 protein interaction network, based on the homologous proteins of *Arabidopsis*, the interacting proteins of CaGH3 were predicted using the STRING database (http://string-db.org/cgi, accessed on 28 July 2024).

### 4.10. Gene Cloning and Sequence Analysis of CaGH3-5 and CaGH3-7

*CaGH3-5* and *CaGH3-7* were chosen, and the full lengths of their coding sequences were cloned from the pepper leaves with primers (Supplementary Table S6) using 2X Xerox PCR Master Mix (Biomed, Beijing, China). The PCR products were purified with a gel extraction kit (Tiangen, Beijing, China), ligated into the pEASY^®^- T1 simple cloning vector (TransGen, Beijing, China), and sequenced. The ClustalX 1.83 and ESPrit3.0 online software (http://espript.ibcp.fr/ESPript/cgi-bin/ESPript.cgi, accessed on 13 May 2022) were used for multiple alignments of the amino acid sequences of proteins in group I of the phylogenetic tree.

### 4.11. Subcellular Localization of CaGH3-5 and CaGH3-7

The full-length coding sequences of CaGH3-5 and CaGH3-7 were separately amplified and inserted at the N-terminal of GFP of pCAMBIA1300 with the primers (Supplementary Table S6). Then, the GV3101 strains harboring CaGH3-5/CaGH3-7-YFP or 35S::YFP were transformed into 5-week-old *Nicotiana benthamiana* leaves [59]. GFP signals were analyzed 72 h after infiltration via confocal microscopy. PIP2-mCherry was used as a membrane localization marker [60].

### 4.12. Vector Construction, Plant Transformation, and Transcriptome Analysis

The full coding sequence of *CaGH3-7* was cloned into the plant expression vector PHG using BamH I and Pst I restriction sites to generate 35S::*CaGH3-7* with the primers (Supplementary Table S6). The vector was transformed into the *Agrobacterium tumefaciens* strain *GV3101* and then transformed into Micro-Tom [61]. Fifty-day-old seedlings from three independent lines of the wild type and 35S:: *CaGH3-7* were used for RNA-Seq analysis.

A total of six mRNA libraries were prepared according to the Illumina RNA sequencing protocols and sequenced using paired-end sequencing with 150 bp lengths on the NovaSeq X plus platform (Illumina, San Diego, CA, USA). The RNA-Seq data have been uploaded onto the China National Center for Bioinformation database under designation number PRJCA034753. Genes with |log2 fold change| ≥ 1 and a false discovery rate <0.05 were termed differentially expressed genes (DEGs) according to the following comparison: 35S::*CaGH3-7* vs. WT. DEGs were classified and grouped using Gene Ontology (GO, http://geneontology.org/, accessed on 6 February 2025) and Kyoto Encyclopedia of Genes and Genomes (KEGG, http://www.genome.jp/kegg/, accessed on 1 January 2025) analyses to identify the associated biological pathways. The significance threshold was set at *p* < 0.05.

## 5. Conclusions

This study conducted a genome-wide analysis of the *GH3* gene family in pepper, and a total of eight *CaGH3* genes, distributed on four chromosomes and classified into two groups (I and III), were identified based on bioinformatic analysis. They all had a conserved domain and close evolutionary relationships with *S. lycopersicum*. Many cis-elements related to plant hormones and stress were distributed in the promoter regions of the *CaGH3* genes. Furthermore, the *CaGH3* genes were differentially expressed among pepper tissues and could be induced by phytohormones (IAA, ABA, and MeJA) and abiotic stresses (salt, low temperature, and drought). This indicates that they participated in various phytohormones and stress-signaling pathways. *CaGH3-5* and *CaGH3-7* were cloned and both located in the membrane and chloroplast. Notably, after overexpressing *CaGH3-7* in tomato, differential expressed genes were mainly enriched in response to external stimuli. Our data may help in screening genes for further functional identification and genetic improvement, which is beneficial to accelerating the process of pepper resistance breeding.

## Figures and Tables

**Table 1:** Basic characteristics of 8 *CaGH3* genes in *Capsicum annuum*.

Gene Name	Gene ID	Protein Length (aa)	Molecular Weights (KDa)	Isoelectric Point (pI)	Instability Index	Hydrophilic Coefficient	Chromosome	Subcellular Localization	α-Helix (%)	β-Turn (%)	Extended Strand (%)	Random Coil (%)
*CaGH3-1*	Capana02g000676	602	68.04	6.07	40.99	−0.24	2	Chloroplast	42.86	5.15	14.45	37.54
*CaGH3-2*	Capana02g003021	599	67.71	5.48	43.18	−0.23	2	Chloroplast	41.74	5.01	13.69	39.57
*CaGH3-3*	Capana07g001573	605	68.58	5.39	40.33	−0.27	7	Chloroplast	41.65	4.46	13.06	40.83
*CaGH3-4*	Capana07g001662	599	68.34	6.21	40.53	−0.34	7	Chloroplast	43.57	4.51	14.69	37.23
*CaGH3-5*	Capana08g001036	575	64.28	5.67	31.81	−0.14	8	Chloroplast	44.35	4.70	14.43	36.52
*CaGH3-6*	Capana08g002278	595	67.47	6.27	43.75	−0.28	8	Chloroplast	41.85	4.20	13.95	40.00
*CaGH3-7*	Capana10g000405	609	69.42	7.26	47.34	−0.29	10	Chloroplast	43.19	4.93	13.30	38.59
*CaGH3-8*	Capana10g000854	579	64.73	5.54	35.22	−0.11	10	Chloroplast	41.62	4.49	15.54	38.34

## Data Availability

All data are available within the manuscript. The RNA-Seq data have been uploaded onto the China National Center for Bioinformation database under designation number PRJCA034753.

## Supplementary Materials

The following supporting information can be downloaded at: https://www.mdpi.com/article/10.3390/plants14142231/s1, Supplementary Table S1. Total differentially expressed genes in the comparison of WT vs. *CaGH3-7* overexpression in *Solanum lycopersicum*. Supplementary Table S2. GO enrichment analysis of differentially expressed genes. Supplementary Table S3. KEGG pathway enrichment analysis of differentially expressed genes. Supplementary Table S4. Information about the *GH3* genes in various plants. Supplementary Table S5. Predicted conserved motifs of CaGH3 proteins. Supplementary Table S6. Primers used in this study.

## Abbreviations

The following abbreviations are used in this manuscript:

GH3	Gretchen Hagen 3
Aux	Auxin
IAA	Indole-3-acetic acid
SAUR	Small auxin up RNA
SA	Salicylic acid
JA	Jasmonic acid
ABA	Abscisic acid
HMM	Hidden Markov Model
MeJA	Methyl jasmonate
PEG-6000	Polyethylene glycol-6000
MW	Molecular weight
pI	Isoelectric point
ARR	Arabidopsis response regulator
APRR	Arabidopsis putative response regulator-like
GO	Gene Ontology
KEGG	Kyoto Encyclopedia of Genes and Genomes
MAPK	Mitogen-activated protein kinase
